# Editorial: Perception, cognition, and working memory: interactions, technology, and applied research

**DOI:** 10.3389/fpsyg.2023.1190194

**Published:** 2023-04-17

**Authors:** Hong Xu, Edwin James Burns

**Affiliations:** ^1^Psychology, School of Social Sciences, Nanyang Technological University, Singapore, Singapore; ^2^Department of Psychology, Edge Hill University, Ormskirk, United Kingdom

**Keywords:** perception, cognition, attention, memory, action, human computer interaction

## Research questions

The research questions addressed in this Research Topic span from our senses to perception to cognition and behavior, as well as theoretical and mathematical models to explain such processes. In particular, the studies have helped answer questions in visual perception (Bernardinis et al.; Orima and Motoyoshi; Wu et al.; Vargas and Moreno-Ríos), working memory (Liu et al.; Wang A. et al.; Zacharov et al.), attention (Kandana Arachchige et al.), detection (Branch et al.), emotion (Liang et al.), memory (Guo et al.; Chén et al.; Zhou et al.; Setti et al.), auditory processing (Fan et al.), language (Wang Y. et al.; Cui et al.; Zhu and Aryadoust), cognition (Krupitzer et al.; Ren et al.; Shi), actions (Bitu et al.; Zhu et al.; Lukashova-Sanz et al.; Ren et al.), and human-machine interactions (Loriette et al.; Zhang et al.).

## Methods

To address the research questions in the above fields, the studies in our Research Topic used multiple research methods, such as psychophysical experiments (Vargas and Moreno-Ríos; Shi), behavioral tasks (Bitu et al.; Lukashova-Sanz et al.), survey questionnaires (Zhu et al.) and virtual reality simulations (Krupitzer et al.; Lukashova-Sanz et al.). They also used eye tracking to record the participants' eye movement patterns (Wang Y. et al.; Zacharov et al.), and revealed the underlying neural mechanisms through EEG (Liang et al.; Orima and Motoyoshi; Cui et al.; Shi) or its connectivity by transcranial direct current stimulation (tDCS) (Wu et al.).

## Theories

Given the complexity of the tasks, it is a challenge to find an overarching unified theory to explain underlying mechanisms. By contrast, existing theories often explain a narrower collection of data derived from tasks. The current issue tries therefore to collate papers that help face down such challenges, bringing them to our attention, and thus provides an outlet for such research to be published. For example, cognitive load theory (Sweller, 1988; Mayer and Moreno, 2003; Sweller et al., 2011; Bitu et al.; Zhu and Aryadoust) explains how limited amount of cognitive resources are used for a task. Flexible resource (attention) theory (Sandry et al., 2014; Sandry and Ricker, 2020; Zhou et al.) argues that attention resources are allocated flexibly to items in working memory and that their allocation to one item is at the cost of the others. These theories suggest that we have limited capacity in attention, cognitive resources, and working memory (Miller, 1956). This Research Topic also brought up a mathematical modeling for priming (Chén et al.). However, there is still a lack of theories for brain-computer interfaces (Loriette et al.), especially for the connections among sensation, perception, cognition, memory and action.

## Applications

In addition to neurotypical participants, these methods are beneficial for people at a disadvantage, such as the blind and visually impaired (Setti et al.) or those with Parkinson's disease (Bernardinis et al.). Similarly, they may also help provide practical support to those with autism spectrum disorder (Zacharov et al.). They cover studies that tested children (Zacharov et al.; Bitu et al.), soccer players (Ren et al.; Krupitzer et al.), college students (Ren et al.), adults (e.g., Branch et al.) and older adults (Zhu et al.). Setti et al. showed that the blind participants exhibited impaired performance in a spatial memory task of sound locations, adapted from the card game “Memory,” thus confirming “the pivotal role of visual experience in the active manipulation of memorized spatial information.” This suggests that auditory spatial memory benefited from visual integration. While auditory spatial memory of the location is affected by visual experience, it might also be interesting to test participants' responses to tasks that do not require any visual cues of the space, and to examine if the increased sensitivity of one sensory modality is at the cost of the other(s) or enhanced by the other(s).

## Future research

Complex tasks and concepts can often be dissected into distinct steps that occur sequentially or simultaneously. Based on existing theories of limited cognitive resources, the former may be more reasonable, but it also depends on the time scale of this analysis. In a fine grained time-scale, such as milliseconds, it is more likely to be sequential than simultaneously. Different research methods may be able to address the tasks from various perspectives. Therefore, future research may call into the integration of these perspectives, and the underlying mechanisms and theories.

## Author contributions

HX drafted the manuscript. EB provided critical revisions. Both authors approved the final version of the manuscript for submission.
